# Present and future self in memory: the role of vmPFC in the self-reference effect

**DOI:** 10.1093/scan/nsab071

**Published:** 2021-06-18

**Authors:** Debora Stendardi, Francesca Biscotto, Elena Bertossi, Elisa Ciaramelli

**Affiliations:** Department of Psychology, University of Bologna, Bologna 40126, Italy; Center for Studies and Research of Cognitive Neuroscience, Cesena 47521, Italy; Center for Studies and Research of Cognitive Neuroscience, Cesena 47521, Italy; Department of Psychology, University of Bologna, Bologna 40126, Italy; Center for Studies and Research of Cognitive Neuroscience, Cesena 47521, Italy

**Keywords:** self, memory, mental time travel, future thinking, ventromedial prefrontal cortex

## Abstract

Self-related information is remembered better than other-related information (self-reference effect; SRE), a phenomenon that has been convincingly linked to the medial prefrontal cortex. It is not clear whether information related to our future self would also have a privileged status in memory, as medial prefrontal cortex (mPFC) regions respond less to the future than to the present self, as if it were an ‘other’. Here we ask whether the integrity of the ventral mPFC (vmPFC) is necessary for the emergence of the present and future SRE, if any. vmPFC patients and brain-damaged and healthy controls judged whether each of a series of trait adjectives was descriptive of their present self, future self, another person and that person in the future and later recognized studied traits among distractors. Information relevant to the present (*vs* future) was generally recognized better, across groups. However, whereas healthy and brain-damaged controls exhibited strong present and future SREs, these were absent in vmPFC patients, who concomitantly showed reduced certainty about their own present and anticipated traits compared to the control groups. These findings indicate that vmPFC is necessary to impart a special mnemonic status to self-related information, including our envisioned future self, possibly by instantiating the self-schema.

## Introduction

We often find ourselves thinking about who we are: whether we are introvert and why, what are our music preferences, favorite clothes, places, philosophers, what is that drives us crazy or that instead we wish for the future. Instances of self-knowledge such as these revolve around the self-schema, an articulated set of beliefs about oneself, generally deriving from the repeated categorization and subsequent evaluation of one’s behavior, which defines our identity and biases the way we process incoming information (Markus, 1977). Self-knowledge (e.g. ‘I am an introvert person’) is at the border between episodic memory, our ability to recollect personal experiences within their unique spatio-temporal context (e.g. ‘yesterday at the party I only talked to Francesca’), and semantic memory, our (culturally shared) knowledge of facts and concepts by now detached from the context of acquisition (e.g. ‘Introvert does not mean shy’), as it is at the same time personal and devoid of context (Renoult *et al.*, 2012). Self-knowledge is dissociated from episodic and semantic memory. For example, patients with episodic amnesia due to medial temporal lobe (MTL) damage typically have preserved self-knowledge (Klein *et al.*, 1996; Klein and Lax, 2010; Picard *et al.*, 2013), and self-relevant semantic concepts can be preserved in semantic dementia (Westmacott *et al.*, 2001). Self-knowledge is also dissociated from other personal semantic information, such as repeated events characterizing lifetime periods (e.g. ‘In high school, I would hang out only with Francesca’), which are associated with greater contextual detail, and often impaired in MTL amnesia (St-Laurent *et al.*, 2009).

What are the neural bases of self-knowledge? One way to investigate this is to study the footprints self-knowledge leaves on new learning. Rogers *et al.* (1977) found that trait adjectives processed in relation to the self (e.g. ‘are you an introvert person?’) are remembered better than trait adjectives processed for their phonetic or structural properties (e.g. ‘does introvert rhyme with disconcert?’), their meaning (e.g. ‘does introvert mean the same as shy?’), or even in relation to another individual (e.g. ‘is she an introvert person?’; Klein and Kihlstrom, 1986; Symons and Johnson, 1997; Kelley *et al.*, 2002)—a phenomenon called ‘self-reference effect’ (SRE; Rogers *et al.*, 1977). In a functional magnetic resonance imaging (fMRI) study, Kelley *et al.* (2002) found that the medial prefrontal cortex (mPFC) was selectively activated in the self-related condition and not in the other-related or lexical conditions (see also Craik *et al.*, 1999). Macrae *et al.* (2004) showed that activity in the mPFC predicted both judgments of self-relevance for trait adjectives and the SRE in memory, and Kim and Johnson (2012) extended the finding to objects owned by the participants, which were associated with increased subjective value, memorability and mPFC engagement compared to other people’s objects. Together, these findings point to medial prefrontal regions as implicated in self-related processing—a finding that has been corroborated by several meta-analyses (Denny *et al.*, 2012; see also Northoff *et al.*, 2006; Lieberman, 2010; Murray *et al.*, 2012 for reviews), which point to Brodmann’s area (BA) 10 as the most prominent cluster of self-related activity (Lieberman *et al.*, 2019). Consistent with this, patients with mPFC lesions (centered on BA 10) were found to not show the SRE in memory (Philippi *et al.*, 2012).

We do not just reflect on how we are currently, but also on how we were in the past or predict we will be in the future. Thinking about the future shares component processes with remembering the past (Buckner and Carroll, 2007; Schacter *et al.*, 2012) and is as fractionated a process as is remembering the past (Addis *et al.*, 2007; D’Argembeau and Mathy, 2011; D’Argembeau, 2020). For example, patients with MTL amnesia cannot imagine specific future events but can report semantic (including autobiographical) information about the future (Race *et al.*, 2011) and can think about (Kwan *et al.*, 2013) and self-project into the future in abstract terms (Arzy *et al.*, 2009). An important question is how we represent our past and future selves. D’Argembeau *et al.* (2008) asked participants to reflect on their current traits, their traits in the past, and on the current and past traits of another individual. They found that both ventral mPFC (vmPFC) and dorsal medial prefrontal cortex were more active when individuals reflected on their current *vs* past selves and that there was no difference in medial prefrontal activity between the past-self and the ‘other’ condition, as if the past self were perceived, to some extent, as another individual, due to the perceived change, with time, in one’s characteristics, activities and goals (Libby and Eibach, 2002; Pronin and Ross, 2006). Similarly, Ersner-Hershfield *et al.* (2009) found diminished vmPFC activity for the future *vs* present self (see also D’Argembeau *et al.*, 2010), and a recent study confirmed that vmPFC activity while reflecting on our future self in 10 years is more similar to that observed while we think to another individual compared to our current self (Mitchell *et al.*, 2011). None of these studies, however, has investigated whether or not the future self also has a privileged status in memory, and, in case it does, whether the future SRE would also be mediated by mPFC regions.

The aim of the present study is two fold. First, we wish to confirm that the vmPFC is a crucial substrate of self-knowledge, showing that vmPFC damage is associated with a reduced SRE (as in Philippi *et al.*, 2012). There are several reasons to think that vmPFC is related to the SRE. This region is commonly activated during tasks requiring self-reflection (Jenkins *et al.*, 2008; Wagner *et al.*, 2012), and vmPFC patients are impaired in self-monitoring (Beer *et al.*, 2006; Hiser and Koenigs, 2018) and reportedly unable to introspect and daydream (Ackerly and Benton, 1948; Wheeler *et al.*, 1997; Bertossi and Ciaramelli, 2016). Additionally, vmPFC patients have been found to use fewer self-references than healthy and brain-damaged controls while narrating personal events, as if they failed to fill constructed experience with self-related content (Kurczek *et al.*, 2015).

Second, we investigated whether vmPFC is a crucial underpinning of future self-knowledge, by additionally testing whether items related to the future self also give rise to an SRE in memory, and whether the future SRE, too, depends on vmPFC integrity. Previous neuropsychological work has shown that vmPFC damage impairs several components of future thinking, such as the ability to imagine specific future events (Bertossi *et al.*, 2016a,b, 2017; Verfaellie *et al.*, 2019) and also to self-project into future time periods in more abstract terms (Fellows and Farah, 2005; Sellitto *et al.*, 2010; Ciaramelli *et al.*, 2021). However, vmPFC patients can normally report on semantic facts about their personal future (e.g. ‘In my 70s I will be retired’; Bertossi *et al.*, 2016a,b, 2017; Verfaellie *et al.*, 2019), suggesting that future personal semantics, including knowledge about one’s future self, may be retained in these patients. The fMRI evidence that vmPFC responds less to the future than to the present self (Ersner-Hershfield *et al.*, 2009; D’Argembeau *et al.*, 2010) also leads to the prediction that vmPFC patients, compared to the controls, would have an impaired representation of their present self, but not necessarily of their future self. fMRI evidence, however, is correlational in nature, and, therefore, lesion studies are necessary to clarify the functional interpretation of brain activity and its relation to behavior. To this aim, we had vmPFC patients and brain-damaged and healthy controls judge whether each of a series of trait adjectives was descriptive of their present self, future self, another person and that person in the future and then to recognize them among distractors. If the representation of the future self is similar, to some extent, to that of another person (Parfit, 1971; Pronin and Ross, 2006), then the future SRE should have a smaller magnitude compared to the present SRE. Moreover, based on fMRI evidence (Ersner-Hershfield *et al.*, 2009; D’Argembeau *et al.*, 2010), we predict that vmPFC patients would show a reduced present SRE (as in Philippi *et al.*, 2012) but a normal future SRE.

Finally, we sought to begin to shed light on the cognitive bases of the SRE and on possible reasons of SRE anomalies in vmPFC patients. Self-referenced (as opposed, for example, to phonetic) item processing is thought to lead to deep encoding. This is because incoming information is evaluated against the self-schema: participants compare trait adjectives with their self-view. This comparison can have variable epistemic and emotional consequences. Participants may be more or less certain that they possess (or not) a given trait (the ‘epistemic investment’ in the self-view, to say it with D’Argembeau *et al.*, 2012), which depends on the amount and consistency of information one has about this aspect of the self in the self-schema (Pelham, 1991), and they may place more or less importance on having (or not) a trait (the ‘emotional investment’), which reflects the extent to which the trait is related to one’s personal goals and motives (Pelham, 1991). The vmPFC is implicated in schema-related processing (Ghosh *et al.*, 2014) and deemed to generate confidence signals based on the match between incoming information and the self-schema (Hebscher and Gilboa, 2016). vmPFC is also known for its role in emotion and valuation (Lieberman *et al.*, 2019). D’Argembeau *et al.* (2012) indeed found that left BA 10 tracked the certainty of having a trait and right BA 10 tracked its perceived importance, suggesting that vmPFC represents the epistemic and emotional value of trait items. One possibility, therefore, is that the strength of epistemic and emotional responses to trait adjectives relates to the efficacy with which these items are encoded in memory and that the lack of SRE in vmPFC patients is associated with a reduction of these responses. To test this, we asked participants to judge, for each trait, how certain they were to possess or that they will possess that trait and the importance they attached to it.

## Methods

### Participants

Participants included 15 patients with brain damage and 23 healthy individuals. Patients were recruited at the Centre for Studies and Research in Cognitive Neuroscience, Cesena, on the basis of their lesion site, as documented by MRI or computerized tomography (CT) scans. Seven patients had lesions involving vmPFC (vmPFC patients; 7 males; mean age = 57 years, range = 43–74; mean education = 10 years, range = 5–13; see Table 1 for individual patients’ demographic and neuropsychological data). vmPFC patients’ lesions resulted, in all cases, from the rupture of an aneurysm of the anterior communicating artery. They were bilateral in six cases and right-lateralized in one case. The remaining eight patients had brain lesions that did not involve vmPFC (7 males; mean age = 61, range = 41–74; mean education = 11 years, range = 5–18). Control patients’ lesions were caused by ischemic or hemorrhagic stroke, traumatic brain injury or brain tumor and were in the left hemisphere in three cases and in the right hemisphere in five cases. Lesion sites mainly included the occipital cortex, extending into the occipito-temporal area (six cases) and the fronto-parietal cortex (one case). For one of the eight control patients the lesion description was available but MRI scans were not, and therefore we could not reconstruct precisely the extension of the lesion. There was no significant difference in lesion volume between vmPFC patients and the remaining seven control patients (57 *vs* 33 cc., *P* = 0.18). Included patients were in the stable phase of recovery (at least 3 months post-morbid). The healthy control group comprised 23 participants without neurological or psychiatric history (21 males; mean age = 57, range = 47–74; mean education = 11 years, range = 5–18), which were matched to patients on age, education (F_2,35_ < 0.84; *P* > 0.43 in both cases) and gender balance (χ^2^ < 0.94, *P* > 0.32 in all cases). vmPFC patients’ sample size was based on a previous study on the SRE in vmPFC patients (e.g. Philippi *et al.*, 2012: 6 vmPFC patients, 15 healthy controls and 8 control patients). A somewhat larger *N* was chosen for control participants (23 healthy controls and 8 control patients), based on the average effect size of the SRE (*d* = 0.5) in a meta-analysis of 129 studies (Symons and Johnson, 1997), which required a sample size of *N* = 27 to be replicated (*P* = 0.05) with a statistical power = 0.80. Participants gave written informed consent to participate in the experiment, which was performed in agreement with the 2008 World Medical Association Declaration of Helsinki, and approved by the Bioethical Committee of the University of Bologna and the Ethical Committee of Area Vasta (CEIIAV) of Emilia Romagna.

**Table 1.** Patients’ demographic and clinical data

	vmPFC patients						
	p. 1	p. 2	p. 3	p. 4	p. 5	p. 6	p. 7
Sex	M	M	M	M	M	M	M
Age (years)	53	65	51	43	74	54	60
Education (years)	8	13	13	13	5	8	13
Raven standard matrices (cut-off = 15)	23.25	20	19	23.25	22	28.5	−
Attentional matrices (cut-off = 31)	48.5	35	49.5	42.25	57	54.5	49
Phonemic fluency (cut-off = 17)	27	22	32	21	18	36	20
Semantic fluency (cut-off = 25)	37	36	35	40	34	61	34
Wisconsin Card Sorting Test perseverative errors (cut-off = 42)	41	64*	28	64*	−	87*	38
Short-term memory—Digit span (cut-off = 3.75)	5	5.75	5.75	6.5	5.5	5	2.75*
Short-term memory—Corsi tapping test (cut-off = 3.75)	4.75	4.75	3.5	5.5	4	5.75	2.75*
Long-term memory—Prose passage recall (cut-off = 4.75)	5	12.5	13.5	13	9.2	8.6	5.7
Rey complex figure copy (cut-off = 28.9)	32.5	36	36	36	−	35.5	30.25
Rey complex figure delay (cut-off = 9.5)	6.75	9.9	22	19.5	−	15.75	17.25

*Note*: The table reports, for each patient (p), scores corrected for age, education and gender according to normative samples. For each test, we also report the cut-off score. Scores below the cut-off are considered indicative of impaired performance (corresponding to a percentile < 5), and signaled by an *. Dashes indicate missing data.

### Lesion analysis

**Fig. 1.**
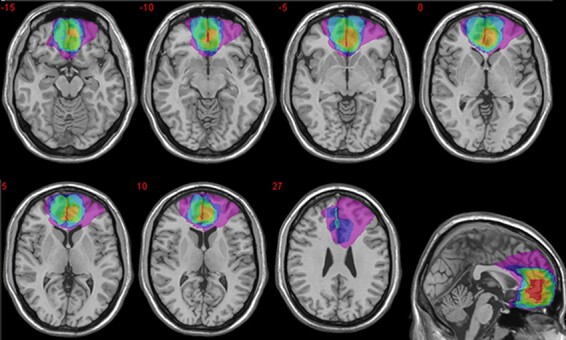
Location and overlap of brain lesions. The panel shows the lesions of the seven patients with vmPFC damage projected on the same seven axial slices and on the mesial view of the standard Montreal Neurological Institute brain. The level of the axial slices is indicated by white horizontal lines on the mesial view of the brain, and by *z*-coordinates. The color bar indicates the number of overlapping lesions. Maximal overlap occurs in BA 11, BA 10 and BA 32 of vmPFC. In axial slices, the left hemisphere is on the left side.

Patients’ individual lesions derived from the most recent MRI or CT scans were manually drawn by a trained neuroscientist directly on each slice of the normalized T1-weighted template MRI scan from the Montreal Neurological Institute distributed with MRIcro (Rorden and Brett, 2000). The MRIcro software was used to estimate lesion volumes (in cc) and generate lesion overlap images. Figure 1 shows the extent and overlap of brain lesions in vmPFC patients. The mainly affected BAs were BA 10, BA 11, BA 24, BA 25 and BA 32, although one patient also had damage to lateral prefrontal regions involving BA 9, BA 46 and BA 47, which accounted for 4–9% of his total lesion size. The region of maximal lesion overlap occurred in BA 11 (*M* = 21.51 cc, s.d. = 8.79), BA 10 (*M* = 12.93 cc, s.d. = 5.35) and BA 32 (*M* = 8.41 cc, s.d. = 4.33).

### Neuropsychological assessment

Patients’ general cognitive functioning was preserved, as indicated by the scores they obtained in the Raven Standard Matrices (Spinnler and Tognoni, 1987), which were on average within the normal range and comparable across participant groups (vmPFC patients: *M* = 23, range: 19–29; control patients: *M* = 22, range: 11–25; healthy controls: *M* = 28, range: 13–39; F_1,31_ = 2.98; *P* = 0.065; Capitani and Laiacona, 1988). vmPFC patients also received a more extensive neuropsychological evaluation, aimed at specifying their cognitive profile further. Table 2 portrays individual vmPFC patients’ scores in standardized neuropsychological tests. vmPFC patients attained normal scores in tests assessing attentional skills (Attentional Matrices; Spinnler and Tognoni, 1987), verbal and spatial short-term memory (Digit Span, Corsi test; Spinnler and Tognoni, 1987), and verbal long-term memory (Prose passage recall test; Spinnler and Tognoni, 1987). As for executive functioning, both phonemic and semantic fluency were within the normal limits (Spinnler and Tognoni, 1987), but a few cases exhibited impaired cognitive flexibility, as apparent in an increased number of perseverative errors in the Wisconsin Card Sorting Test (Heaton *et al.*, 2000).

**Table 2.** Mean recognition accuracy by participant group and encoding condition

	Present-Self	Present-Other	Future-Self	Future-Other	Standard
vmPFC patients	0.11 (0.09)	0.12 (0.15)	0.06 (0.09)	0.05 (0.16)	0.10 (0.20)
Control patients	0.25 (0.26)	0.03 (0.15)	0.15 (0.24)	0.08 (0.19)	0.18 (0.16)
Healthy controls	0.48 (0.17)	0.27 (0.14)	0.43 (0.17)	0.25 (0.16)	0.43 (0.15)

The values in parenthesis are s.d. values.

### Task procedure

A set of 180 adjectives reflecting psychological traits (90 with a positive connotation and 90 with a negative connotation; e.g. sincere and cynic) was selected from Anderson’s (1968) list and translated to Italian. Ninety adjectives were used in the initial rating phase and served as studied items in the following recognition phase, whereas the remaining 90 adjectives served as distractors in the recognition phase. The assignment of trait adjectives to the different rating conditions or to the distractor status (in the recognition phase) was counterbalanced across participants.

In the rating phase, participants were presented with 90 adjectives (half positive and half negative) and were required to make different types of judgment depending on the experimental condition, namely, assess whether the adjective described their current psychological traits (Present-Self condition; 18 items), their anticipated psychological traits in 10 years (Future-Self condition; 18 items), the current psychological traits of Gerry Scotti, a famous Italian showman of approximately the same age as our participants (Present-Other condition; 18 items), and the anticipated psychological traits of Gerry Scotti in 10 years (Future-Other condition; 18 items). We also included a Standard condition (18 items), in which participants judged whether or not the adjective referred to a positive psychological trait, which involves semantic processing but not reflecting on the characteristics of a particular person (self *vs* other). Each trial started with a fixation cross shown for 500 ms. Then a trait adjective appeared, along with the question pertaining to the relevant rating condition (e.g. in the Present-Self condition: how well does this trait describe ‘you now’?), which was written right above the adjective and remained on the screen until the end of the trial. Across conditions, participants responded using a Likert scale from 1 (not at all) to 4 (totally), with no time limit for responding. Participants evaluated different adjectives in each rating condition (counterbalanced), and the order of trials pertaining to the different conditions was randomized for each participant.

About 15 minutes after the rating phase, which were filled with unrelated activities (the Raven task and demographic questionnaires), participants underwent an unanticipated recognition memory task (recognition phase), in which the 90 previously rated adjectives were presented again, but this time intermixed with 90 new trait adjectives. Each trial started with a fixation cross shown for 500 ms. Then subjects were presented with an adjective and had to state whether they remembered it from the previous session or not (old/new judgment).

Finally, subjects were presented again with the trait adjectives they had previously evaluated with reference to the present and future self, and asked to report, for each trait, how certain they were that they possessed (or not) that trait (for items in the Present-Self condition; 18 items) or that they will possess (or not) that trait (for items in the Future-Self condition; 18 items) (epistemic response; D’Argembeau *et al.*, 2012), and how important it was to them that they possessed (or not) that trait (Present-Self condition) or that they will possess (or not) that trait (Future-Self condition) (emotional response; D’Argembeau *et al.*, 2012). In all cases, participants responded using a Likert scale from 1 (not at all) to 4 (totally).

## Results

### Rating (encoding) phase

We first investigated whether there were group differences in the time participants needed to evaluate trait adjectives across experimental conditions (Present-Self, Future-Self, Present-Other, Future-Other and Standard) and in the degree to which participants attributed psychological traits to the self (Present-Self and Future-Self conditions) or to another person (Present-Other and Future-Other conditions), or felt that a personality trait was positive (Standard condition). An analysis of variance (ANOVA) on response times with Group (vmPFC patients, control patients and healthy controls) and Condition (Present-Self, Future-Self, Present-Other, Future-Other and Standard) as factors revealed no significant effects or interaction (*P* > 0.13 in all cases), meaning that participant groups took a similar time to evaluate trait adjectives at encoding, which did not differ across encoding conditions. Because ratings were in some cases non-normally distributed (Kolmogorov–Smirnov *d* > 0.20, *P* < 0.01), the data were analyzed with non-parametric statistics. We found no significant group differences in mean ratings across conditions (Median test χ2 < 4.84, *P* > 0.08 in all comparisons). We obtain similar findings by analyzing positive and negative personality traits separately.

### Recognition phase

**Fig. 2.**
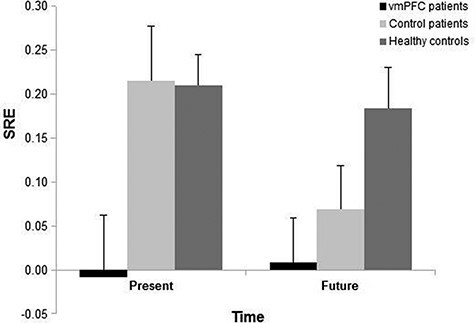
The SRE relative to the present and the future in vmPFC patients, and healthy and brain-damaged controls. Bars indicate SEM values.

Table 2 shows mean accuracy (hit rates − false alarm rates) by participant group and rating condition (Present-Self, Future-Self, Present-Other, Future-Other and Standard), and Figure 2 shows the SRE relative to the present and the future by participant group. We obtained a similar pattern of results analyzing recognition accuracy for positive and negative traits separately, and so, for clarity, we report on the collapsed results.

#### Standard recognition accuracy.

As a preliminary assessment of general recognition memory abilities across participant groups, we conducted a one-way ANOVA on recognition accuracy in the Standard condition with Group (vmPFC patients, control patients and healthy controls) as factor. The ANOVA revealed a significant effect of Group (F_2,35_ = 13.70, *P* = 0.00004, η^2^_p_ = 0.44). *Post hoc* comparisons, conducted with the Duncan test, showed that both vmPFC (0.10 *vs* 0.43, *P* = 0.0002) and control patients (0.18 *vs* 0.43, *P* = 0.002) had lower recognition accuracy compared to healthy controls, but there was no significant difference in recognition accuracy between vmPFC patients and control patients (*P* = 0.32; Table 2).

#### Recognition accuracy for self and other present and future traits.

We next investigated the effect of self-reference and time on recognition accuracy. We ran a three-way ANOVA on recognition accuracy with Group, Self-reference (Self and Other) and Time (Present and Future) as factors. The ANOVA revealed a significant effect of Time (F_2,35_ = 5.89, *P* = 0.02, η^2^_p_ = 0.14), indicating that trait adjectives evaluated with reference to the present were generally recognized better than those evaluated with respect to the future. Moreover, there were a significant effect of Group (F_2.35_ = 16.66, *P* < 0.00001, η^2^_p_ = 0.49) and a significant effect of Self-reference (F_2,35_ = 16.85, *P* < 0.0001, η^2^_p_ = 0.83), qualified by a Group × Self-reference interaction (F_2,35_ = 4.73, *P* = 0.015, η^2^_p_ = 0.21). *Post hoc* comparisons confirmed that patients’ recognition accuracy in the Other-conditions was significantly (in the case of control patients: 0.059 *vs* 0.26; *P* = 0.038) or numerically lower (in the case of vmPFC patients: 0.085 *vs* 0.26; *P* = 0.06) than that of healthy controls, while their recognition accuracy in the Self-conditions was significantly lower than that the controls (control patients: 0.20 *vs* 0.46; *P* = 0.006; vmPFC patients: 0.085 *vs* 0.46; *P* = 0.0002). There were no differences, however, in recognition accuracy between vmPFC patients and control patients in either the Other-conditions (*P* = 0.76) or the Self-conditions (*P* = 0.17). Crucially, whereas healthy controls (0.46 *vs* 0.26; *P* < 0.0003) and control patients (0.20 *vs* 0.06; *P* = 0.008) evinced higher recognition accuracy when evaluating adjectives with reference to the self than to the other, no such modulation was observed in vmPFC patients (0.085 *vs* 0.085; *P* = 1), who attained a similar recognition accuracy in the Self- *vs* Other-conditions, thus showing no SRE. There were no other significant effects (*P* > 0.1 in all cases) (Table 2).

#### Present and future self-reference effect.

To quantify the SRE (or lack of) directly, we computed an SRE index by subtracting accuracy in the Other-condition from that in the Self-condition, separately for the Present and Future time. An ANOVA performed on the SRE, with Group and Time as factors, showed a significant effect of Group (F_2,35_ = 4.72, *P* = 0 0.015, η^2^_p_ = 0.21), indicating a reduced (virtually absent) SRE in vmPFC patients compared to healthy controls (0.00 *vs* 0.20; *P* = 0.008) and control patients (0.00 *vs* 0.14; *P* = 0.04), with no difference between the control groups (*P* = 0.43). There were no other significant effects (*P* > 0.22 in all cases). The effect of Group remained significant when we inserted (baseline) recognition accuracy in the Other-conditions (i.e. collapsing across the Present-Other and Future-Other conditions) as a covariate (F_2,34_ = 8.15, *P* = 0.001, η^2^_p_ = 0.32), indicating that the SRE was reduced in vmPFC patients compared to both healthy controls (*P* = 0.005) and control patients (*P* = 0.03), with no difference between the control groups (*P* = 0.40). The effect of the covariate was also significant (F_1,34_ = 5.92, *P* = 0.02, η^2^_p_ = 0.14), such that participants with the lowest performance in the Other-conditions were those that enjoyed the greatest SRE (β = −0.27). No other effect was significant (*P* > 0.27 in all cases).

### Certainty and importance ratings of self traits

To begin investigating possible cognitive factors associated with the lack of SRE in vmPFC patients, we analyzed the certainty and importance ratings they gave to personality traits. Table 3 shows mean certainty and importance ratings by participant group and condition. An ANOVA on certainty ratings with Group and Time as factors showed a significant effect of Group (F_2,35_ = 5.22, *P* = 0.01, η^2^_p_ = 0.22), indicating that vmPFC patients were less certain to possess (or not) given personality traits compared to both healthy controls (2.64 *vs* 3.05; *P* = 0.01) and control patients (2.64 *vs* 3.25; *P* = 0.001), with no difference between the control groups (*P* = 0.26). There was also a significant effect of Time (F_1,35_ = 22.32, *P* = 0.00003, η^2^_p_ = 0.39), such that all participants reported they were less certain about the traits they anticipated they might possess (or not) in the future compared to those they thought they had (or not) now (2.90 *vs* 3.12). The Group × Time interaction was not significant (*P* = 0.64). The same ANOVA on importance ratings evinced an effect of Group (F_2,35_ = 3.77, *P* = 0.03, η^2^_p_ = 0.17), indicating that vmPFC patients attributed less importance than healthy controls to possessing (or not) given personality traits (2.73 *vs* 3.13; *P* = 0.03), but their importance ratings were similar to those of control patients (2.73 *vs* 2.87; *P* = 0.12). There was no difference between the control groups (*P* = 0.43). No other effect in the ANOVA was significant (*P* > 0.18 in all cases).

**Table 3.** Mean certainty and importance attributed to self traits by participant group and time condition

	Certainty ratings		Importance ratings	
	Present-Self	Future-Self	Present-Self	Future-Self
vmPFC patients	2.79 (0.46)	2.48 (0.36)	2.81 (0.43)	2.67 (0.48)
Control patients	3.34 (0.58)	3.15 (0.51)	2.81 (0.41)	2.94 (0.44)
Healthy controls	3.16 (0.27)	2.95 (0.38)	3.16 (0.34)	3.11 (0.41)

The values in parenthesis are s.d. values.

### Relation between certainty and importance ratings and recognition accuracy: exploratory analyses

We investigated whether recognition accuracy in the Self-conditions was related to certainty and importance ratings to trait adjectives. We ran a linear mixed effect model on single trait adjective data (*N* = 1368) with recognition accuracy as the dependent variable (1 = hit and 0 = miss); Certainty ratings, Group (vmPFC patients, control patients and healthy controls) and Time (Present and Future) as fixed effects; and Subject as a random effect. There were a significant effect of Certainty ratings (χ^2^ = 11.4, *P* = 0.001), such that trait adjectives associated with high certainty ratings were more likely to be correctly recognized, and a significant effect of Group (χ^2^ = 8.72, *P* = 0.01), such that recognition accuracy in the Self-conditions was lower in vmPFC patients compared to healthy (*P* < 0.0001) and brain-damaged controls (*P* = 0.02). No other effect or interaction in the model was significant (*P* > 0.40 in all cases). The same model considering Importance ratings, Group and Time as fixed effects and Subject as a random effect yielded a significant effect of Importance ratings (χ^2^ = 10.88, *P* = 0.001) and a significant effect of Group (χ^2^ = 9.43, *P* = 0.01), qualified by an Importance rating × Group interaction (χ^2^ = 7.78, *P* = 0.02). The interaction indicated that importance ratings predicted recognition accuracy significantly in healthy controls (χ^2^ = 10.23, *P* = 0.02) and in control patients (χ^2^ = 11.19, *P* = 0.01), but not in vmPFC patients (*P* = 0.7).

These findings indicate that, in healthy controls and control patients, recognition accuracy for self-referenced items was related to the certainty and importance participants associated with possessing (or not) given personality traits. Certainty ratings predicted recognition accuracy in vmPFC patients also, whereas importance ratings appeared untied to recognition accuracy in this group. When we ran again the ANOVA on the SRE with Group and Time as factors, this time including certainty and importance ratings (collapsed across the Present-Self and Future-Self conditions) as covariates, the original effect of Group was no longer significant (*P* = 0.16), as were all other effects in the ANOVA (*P* > 0.28 in all cases), which suggests that the reduced SRE observed in vmPFC patients may be related, at least in part, to their reduced epistemic and emotional responses to adjective traits.

## Discussion

This study investigated the recognition memory advantage for items (trait adjectives) referenced to the self *vs* someone else (SRE) and relative to the present *vs* future time in vmPFC patients, control patients and healthy controls. First of all, we confirmed the presence of an SRE in healthy participants, which was abolished in vmPFC patients, in line with the findings obtained by Philippi *et al.* (2012). Moreover, we showed that healthy controls and control patients also exhibit a future SRE, that is, better recognition accuracy in association with traits evaluated against their view of themselves (*vs* another individual) in the future. The future SRE was, again, absent in vmPFC patients, as was the SRE for the present self. Contrary to our predictions, the present and future SREs had comparable magnitude. This was because, across groups, evaluating trait items from a future (as opposed to present) time perspective resulted in lower recognition accuracy, but this held for both self-referenced items and other-referenced items alike, and therefore did not affect the SRE (difference between Self- and Other-conditions).

Before discussing each of these three main findings in turn, we wish to emphasize that the lack of SRE observed in vmPFC patients is not a common consequence of brain damage, for example reflective of a weakened sense of self following illness and perceived vulnerability (Ciaramelli *et al.*, 2019), as it was not observed in control patients (see also Philippi *et al.*, 2012). It is also unlikely to depend on generally poor recognition memory abilities or comprehension of task instructions on the vmPFC patients’ part. Indeed, although vmPFC patients’ recognition accuracy was worse than that of healthy controls across conditions, so was that of control patients, and yet they evinced a normal SRE. Moreover, it does not seem that vmPFC patients failed at distinguishing different task conditions (e.g. Self *vs* Other). Indeed, they showed better memory for items encoded with reference to the present *vs* future time perspective, as did the other groups, suggesting they were normally responsive to the encoding demands.

Our primary finding that the SRE is abolished in vmPFC patients confirms previous evidence that medial prefrontal regions (Philippi *et al.*, 2012), including vmPFC (this study), are crucially linked to the representation of the self. In addition, our study points to the persistence of the SRE when evaluating the future self in healthy controls and control patients and of its absence in vmPFC patients. The evidence of a future SRE suggests that, although fMRI evidence shows lower medial prefrontal activity for the future than for the present self (Ersner-Hershfield *et al.*, 2009; D’Argembeau *et al.*, 2010), our future self is not an ‘other’: what is encoded with reference to the self, whether past or future, is more frequently remembered than what is encoded with respect to others. The absence of a future SRE in vmPFC patients, therefore, reinforces the view of vmPFC as implicated in self-related processing (Northoff and Bermpohl, 2004; Northoff *et al.*, 2006; Moran *et al.*, 2006; Schmitz and Johnson, 2007).

We found that recognition accuracy for self-referenced traits is predicted by the certainty with which individuals think they possess or will possess those traits (or not) and by the importance they attribute to possessing (or not) those traits. This finding suggests that trait items that are more relevant to our self-schema, because they contribute to define ourselves (the way we definitely are and are not and the way we definitely think we will be or not be) and the value we attach to our (present and future) self-views, enjoy a privileged encoding in memory. Importantly, vmPFC patients were less confident about possessing or not possessing certain personality traits compared to healthy and brain-damaged controls, consistent with previous findings of activity in BA 10 in association with the expression of certainty in self-views (D’Argembeau *et al.*, 2012). A possibility, therefore, is that vmPFC patients did not show an SRE because they are less certain about the traits they do or do not possess, or those they will or will not possess, due to a weakened self-schema or schema instantiation (Gilboa *et al.*, 2006; Ghosh *et al.*, 2014). Consistent with this proposal, vmPFC patients are particularly impaired at imagining self- *vs* other-related future events, as if they failed to activate schematic self-knowledge that drives the collection of individual details of events (D’Argembeau and Mathy, 2011; Verfaellie *et al.*, 2019). Moreover, vmPFC is deemed to generate coherent confidence signals based on the evaluation of personal information against the self-schema (Hebscher and Gilboa, 2016). Indeed, vmPFC damage is often associated with confabulation, the production of false memories for (unhappened) events even blatantly inconsistent with the self-schema (Moscovitch, 1995; Gilboa *et al.*, 2006), which are typically held with abnormal conviction (Gilboa *et al.*, 2006; Ciaramelli and Ghetti, 2007). vmPFC patients also attributed less importance to having given traits than controls, and, unlike the control groups’ importance ratings, their importance ratings were not related to recognition accuracy for self-related items. However, vmPFC patients’ importance ratings did not differ from those of control patients, who showed an SRE, and therefore are less likely to underlie vmPFC patients’ lack of SRE. Although our findings indicate that recognition accuracy is related to certainty and importance ratings for self-related trait items, future studies involving more patients are needed to confirm whether the SRE reduction observed in vmPFC patients is critically linked to their reduced certainty (and importance) responses.

An interesting finding of our study is the mnemonic consequence of adopting a future time perspective. We observed, across groups, a decline in recognition accuracy when participants encoded (both self-referenced and other-referenced) information with respect to a future compared to a present time perspective. Why is information belonging to the future remembered less than that belonging to the present? All participant groups reported they were less certain about their traits in the future than in the present. This finding aligns with the ‘failure of imagination theory’, according to which people find it difficult to imagine how their future self will be (Frederick *et al.*, 2009; Hershfield and Bartels, 2018), which appears to extend to others’ future. We propose, therefore, that a less vivid representation of the future (*vs* present) led to relatively shallower trait encoding in both self- and other-referenced conditions, resulting in lower recognition accuracy. The fact that a time (future *vs* present)-dependent modulation of recognition accuracy was observed in vmPFC patients as well controls highlights areas of spared time processing in vmPFC patients. This finding, indeed, indicates that even though vmPFC patients are impaired in imagining specific future events (Bertossi *et al.*, 2016a,b, 2017; Verfaellie *et al.*, 2019), in self-projecting into future time periods (Ciaramelli *et al.*, 2021; see also Sellitto *et al.*, 2010) and also in representing future self-knowledge (this study), they are capable of distinguishing between different time moments, suggesting that vmPFC integrity is not necessary to conceive time in abstract terms. D’Argembeau *et al.* (2010) found that the inferior parietal cortex was more active when participants reflected on their past and future compared to current selves, a pattern of activity opposite to that displayed by vmPFC. One possibility, therefore, is that vmPFC supports self-related processing, but it is the inferior parietal cortex that mediates the representation of time and temporal distances (Bueti and Walsh, 2009; Nyberg *et al.*, 2010).

To conclude, we have confirmed that self-related information is prioritized in memory and found that this mnemonic advantage extends to information that is relevant to the future self. The present and future SREs are crucially linked to vmPFC integrity, as we found them abolished in vmPFC patients, and this was not a common consequence of brain damage or poor recognition memory. Rather, vmPFC patients showed reduced certainty for self-relevant information (their own traits) compared to the control groups, which we interpret as a consequence of a weakened self-schema or schema instantiation. Interestingly, all participants evinced lower recognition accuracy for future-referenced compared to present-referenced items, suggesting that the present, in addition to the self, is prioritized in memory, which was linked, again, to increased certainty in association with present- *vs* future-referenced information. vmPFC patients, too, showed this present-related memory advantage, meaning they can represent different time moments, at least in these abstract, impersonal terms.

## Data Availability

Data that support the findings of this study are available upon reasonable request.
